# Supplementary material for: Content-rich biological network constructed by mining PubMed abstracts
Source: BMC Bioinformatics. 2004 Oct 8;5:147. doi: 10.1186/1471-2105-5-147 (PMC528731; doi:10.1186/1471-2105-5-147)
Supplement: Additional File 2 — The original results of the above study (non-essential files are deleted to keep the file size under the limit set by BMC bioinformatics). [file 1471-2105-5-147-S2.bz2 › chilibotAdditionalFile2/dip05/224ID7831290E740/html/PLCG1_SYK.html]

 


 **PLCG1** and **SYK** 
  
Found 77 abstracts in PubMed, retrieved 05.  
 

 What does Google say? 
 PDF only 
| .edu only 

---

**Interactive relationship** (e.g. stimulation, inhibition, etc)

**Stimulatory relationship**- Activation of src and  **Syk**  kinases plays a significant role in signal transduction of heterophil degranulation probably by stimulating downstream phosphorylation of PLC  [ **PLCG1** ] , PLD, and PI3 K.  Ref: 12757738 Int Immunopharmacol, 2003
**Neutral relationship**

**Non-interactive relationship** (e.g. studied together, co-existance, homology, etc.)

- In B cells from Cbl b deficient mice cross linking the BCRs resulted in sustained phosphorylation of Igalpha,  **Syk** , and phospholipase C PLC  [ **PLCG1** ]  gamma2, leading to prolonged Ca2 mobilization, and increases in extracellular signal regulated kinase ERK and c Jun NH2 terminal protein kinase JNK phosphorylation and surface expression of the activation marker, CD69.  Ref: 12771181 J Exp Med< MedlineTA>J Exp Med, 2003
- Fc varepsilon RI induced tyrosine phosphorylation of phospholipase C gamma PLC  [ **PLCG1** ]  gamma but not  **Syk**  was significantly suppressed in these cells.  Ref: 12810085 Biochem Biophys Res Commun, 2003
- The Src family kinase  **Syk** , the B cell adaptor protein BLNK, and phospholipase PLC  [ **PLCG1** ]  gamma2 were consistently absent from Reed Sternberg cells.  Ref: 12881301 BloodBlood, 2003
- Engaging CRs on chicken heterophils activates a proximal tyrosine kinase src and  **Syk**  kinases >PLC  [ **PLCG1** ]  PLD >PI3 K >JNK signal transduction pathway that induces degranulation.  Ref: 12757738 Int Immunopharmacol, 2003
- In contrast, the tumor cells in all cases of lymphocyte predominance Hodgkin rad s disease were positive for Fyn,  **Syk** , BLNK and PLC  [ **PLCG1** ]  gamma2, and Lyn immunostaining was seen in a minority of biopsies.  Ref: 12881301 BloodBlood, 2003
